# Poisoning by Tincture of Aconite

**Published:** 1870-03

**Authors:** 


*Poisoning by Tincture of Aconite.*—As this preparation, in combination with other agents, is now a favorite remedy with many practitioners in the treatment of alveolar periostitis, &c., and from that fact that it is a poison, it is very essential that all who make use of it should have a knowledge of its properties and the antidotes, where such may be required.

It is not unusual for patients to be supplied with a quantity of the mixture, of which the tincture of aconite is one of the most active ingredients, to enable them to make frequent local applications at their homes, and for convenience the bottle containing it is often kept upon the dressing table, and hence liable to be swallowed in mistake for some innocent preparation.

Hence the necessity for impressing upon our patients the importance of care, both in regard to the use of this agent, and its safety at their homes.

Dr. B. W. Richardson, of London, was lately called in haste to see a lady, aged 25 years, who, while suffering from severe facial neuralgia, swallowed through mistake, instead of a tonic mixture two tablespoonfuls of an aconite mouth lotion, her mouth being at the time benumbed by a previous use of the lotion to the gums. The mixture was swallowed at 11 o'clock A. M., and she visited several stores and did not reach home until half past twelve o'clock P. M., when she became alarmingly ill, staggered on attempting to walk, and was seized with a fearful benumbed tingling in the lower half of the back, then in the face and head, while at the same time the tingling in the mouth became developed. The head felt as if it were distorted by the pressure of a vice, and a sensation of tightness across the nose and eyes was most distressing. In a few minutes more, the legs became so weak, and such tremor came over her, that she could not stand without assistance. She was conveyed immediately to the house of an acquaintance in the neighbourhood, her friends being under the impression that she was too prostrate for the drive home, a much longer distance. She was placed upon a sofa. The debility had become so great that she fainted on three or four occasions in attempting to set up. Benumbed tingling of both the upper and lower extremities commenced at half-past one o'clock P. M., and vision became very imperfect, a blackness, as she described it, having come over the sight. A little time afterwards, vomiting of an olive-yellow-coloured fluid commenced, and was almost incessant up to seven o'clock P. M. Towards evening she was greatly collapsed, and having fainted when in this state, her friends fancied that she had expired."

Dr. Richardson gives the following account of his treatment:

"The pulse was felt with difficulty at the wrists, and the heart's action was weak and irregular. I gave her immediately some warm brandy punch, and, in a few minutes afterwards, a mixture composed of aromatic spirit of ammonia, sulphuric ether, tincture of ginger, and camphor mixture. A sinapism was placed over the heart, and one upon the calf of each leg. The punch and the mixture were not retained upon the stomach.

She was pulseless at ten minutes to seven o'clock, and the extremities were cold as death. The pupils were much dilated. The intellect continued unimpaired.

It being obvious to my mind that death at the heart had commenced, I resolved to inject hypodermically twenty-five drops of liq. ammoniæ; but as the time that would be required to procure my own syringe might be a fatal loss to the patient, I sent to a neighbouring cutler, who was kind enough to send me one in a few minutes.

Seven o'clock P. M., I injected half a drachm of liq. ammoniæ under the skin, corresponding to the insertion of the right deltoid muscle.

Ten minutes past seven o'clock: Vomiting not so frequent; but the stomach will not tolerate the stimulants. She continues collapsed and very cold; forehead covered with sweat, eyes glassy, and pupils are much dilated; tongue pale and contracted; no trace of pulse at the wrists: intellect unimpaired. Injected half a drachm of liq. ammoniæ under the skin of the outside of the right arm, about midway between the elbow and seat of the first injection.

Twenty minutes past seven o'clock; Vomiting at longer intervals; still pulseless at the wrists, and no sign of return of warmth in the extremities; complains constantly of the compressed and distorted feeling of the head; pupils have continued of the same size. Injected half a drachm of liq. ammoniæ under the skin of left infra-scapular region.

Twenty-five minutes past seven o'clock: Pulseless. Injected half a drachm of liq. ammoniæ under the skin a little below the middle of the outer part of the left arm.

Half past seven o'clock : While my fingers were applied over the course of the radial artery, at the wrist, searching for a pulsatiou, I fancied I felt a weak, irregular, thready beating of the vessel. In a few minutes this became no longer doubtful, but gradually stronger and stronger.

Eight o,clock : Pulse fully established, but a little irregular ; vomiting has almost ceased ; extremities warming ; tingling of the skin and compressed sensation of the head and face no longer felt. The tingling, however, of the extremities, although not so decided, did not cease until half-past twelve o'clock next morning ; and that of the lower lip continued until November 28.

In cases in which death is to all appearances impending, I should not like to lose time in trying to limit the injection to the vein, as suggested by Professor Halford, in poison from snake bites, and would rather take the chance of a sufficient quantity of the ammonia being absorbed from the areolar tissue before its local action takes place, the chief objection to this procedure. Of the four injections made under the skin in Miss B.'s case, but one caused subsequent annoyance, the cutaneous eschar that resulted from it being about the size of one of our new halfpennies. There being no doubt that the symptoms were caused by tincture of aconite, the important matter to ascertain was the quantity that had been taken. I therefore made the necessary inquiries on the point, and learned that the lotion, if made according to the directions for compounding it, should have contained one drachm and a half of the tincture in every fluid ounce. Of this lotion Miss B. took two tablespoonfuls, as already mentioned.

Whether or not the late appearance of the symptoms was owing to the tincture being a weak preparation, or to the fact that it had been taken immediately after breakfast, or even to some peculiar idiosyncrasy, are matters for conjecture. At all events, when they were established, they were of the most alarming nature, and portended approaching death."

Large quantities of animal charcoal are also beneficial in cases of aconite poisoning, as the charcoal absorbs the agent readily.

